# Performance of Soil Moisture Sensors at Different Salinity Levels: Comparative Analysis and Calibration

**DOI:** 10.3390/s24196323

**Published:** 2024-09-29

**Authors:** Qiuju Qi, Hai Yang, Quanping Zhou, Xiaole Han, Zhengyang Jia, Yuehua Jiang, Zi Chen, Lili Hou, Shijia Mei

**Affiliations:** 1 Nanjing Center, China Geological Survey, Nanjing 210016, China; qiqiuju@mail.cgs.gov.cn (Q.Q.);; 2 Key Laboratory of Watershed Eco-Geological Processes, Ministry of Natural Resources, Nanjing 210016, China; 3 College of Hydrology and Water Resources, Hohai University, Nanjing 210098, China; 4 MOE Key Laboratory of Groundwater Circulation and Environmental Evolution, China University of Geosciences (Beijing), Beijing 100083, China

**Keywords:** soil dielectric sensor, distortion, salinity level, soil moisture level, calibrated formula

## Abstract

Soil dielectric sensors have been widely used to obtain real-time soil moisture data, which are important for water resource management. However, soluble salts in the soil significantly affect the accuracy of these sensor measurements. Therefore, it is crucial to select suitable soil dielectric sensors for soil moisture measurements at different salinity levels. Eight mainstream sensors (EC-5, 5TE, Teros12, Hydra-probe II, TDR315L, TDR315H, TDR305H, and CS655) were selected and tested at four different soil salinity levels (EC_1:5_ = 3.0, 1.5, 1.0, and 0.75 dS·m^−1^). The measured values using the factory calibration formulas were compared at six soil moisture levels. The results showed that the measured soil moisture values from various sensors exhibited varying degrees of overestimation, which increased with increasing salinity. Only EC-5 did not exhibit distortion at high-salinity levels, with the measured values showing a good linear trend compared to the standard values. Mutational distortion of the measured apparent dielectric permittivity occurred in TDR315L, TDR315H, Hydra-probe II, and 5TE at EC_1:5_ = 3.0 dS·m^−1^. Insensitive distortion of the measured apparent dielectric permittivity occurred in Teros12 and TDR305H at EC_1:5_ = 3.0 dS·m^−1^ as well as in Teros12, TDR305H, 5TE and Hydra-probe II at EC_1:5_ = 1.5 dS·m^−1^. All tested sensors performed reasonably well at EC_1:5_ ≤ 1.0 dS·m^−1^. Seven sensors (excluding CS655) were calibrated within the distortion threshold. The soil moisture accuracy using the calibrated formulas could reach ±0.02 cm^3^·cm^−3^. At EC_1:5_ ≤ 1.0 dS·m^−1^, most sensors in this study could be applied with the factory calibration formulas. TDR series, EC-5, 5TE and Teros12 were recommended after calibration for EC_1:5_ > 1.0 dS·m^−1^. For extremely high soil salinity levels, the TDR series and EC-5 may be the best choices.

## 1. Introduction

Soil water content (moisture) is essential for agricultural management and drought warning, as it indicates the soil water status. Real-time and accurate soil moisture data are very important for studying water resources, crop management, and disaster analysis [1,2]. Soil moisture sensors (SMSs) can be used for real-time and in situ monitoring of soil volumetric water content (VWC) and are widely used in agriculture, hydrology, ecology, geophysics, and the environment, and have been a mainstream technique for many years [2,3,4]. Various techniques for soil moisture measurement, including nuclear magnetic resonance (NMR), neutron activation, resistance and dielectric sensors [5], among which dielectric sensors are popular due to their simplicity and efficient data handling [6,7]. Dielectric sensors use radiofrequency properties, such as capacitor charging time, travel time, oscillation frequency and attenuation, as indicators to estimate soil characteristics. Time Domain Reflectometry(TDR), Frequency Domain Reflectometry(FDR) and capacitance-based sensors are the main dielectric sensors used for monitoring soil moisture [8].

The accuracy of SMSs is affected by various factors, such as soil texture, soil salt content, soil moisture, temperature and organic matter content [9]. Soil salt content has been proven to severely affect the measurement accuracy of SMSs [10,11,12,13]. The transmission of electromagnetic waves is greatly affected under high-salinity conditions, leading to dielectric losses and overestimated soil moisture content [14]. Some previous work showed that when the soil salinity level reached a certain threshold, the measurement results of SMSs were inaccurate. For example, many TDR methods are only suitable for soils with bulk EC values of up to 5 dS·m^−1^ [15]. Leinauer et al. [16] suggested that TDR and FDR sensors could obtain accurate measurements only when the bulk EC value of the soil was below 4 dS·m^−1^. However, others pointed out that the VWC readings of TDR were affected only when the electrical conductivity of the soil solution exceeded 17.0 dS·m^−1^ [17]. Previous work showed that, CS625 [18], CS655 [19], 5TE [20] and other capacitance-based sensors [21] were very sensitive to variations in soil salinity. Some sensors, such as capacitance-based sensors, overestimated the VWCs as the soil salinity increased, e.g., Turf Guard FDR, CS616, 5TE, SM100 and SMEC300 [22,23,24,25]. In contrast, some sensors, such as TDT [22] and Watermark [25], underestimated VWCs as soil salinity increased. Alternatively, the measured VWCs of EC-5 were underestimated when the EC value of the soil solution was 0.8 dS·m^−1^, while they were overestimated when the EC value of the soil solution increased to 2.5 dS·m^−1^ [26,27]. TDR-based sensors were considered insensitive to soil salinity [15,18,19], due to their relatively high dielectric measurement frequencies [28]. Although some sensors have been tested in high-salinity environments, the accuracy and effects of soil salinity on their performance under the same soil conditions have not yet been investigated.

Sensor manufacturers provide factory calibration formulas for specific sensors, which greatly influences the measurement accuracy of the soil water content [2,27,29]. The factory calibration process is usually conducted with homogeneous soil (sand or loam) under standard laboratory settings [30]. However, applying factory calibration formulas to other scenarios with significant differences in temperature, soil type, salinity and other factors could be more reasonable, yielding inaccurate [31] or even unrealistic values [32,33,34]. Evidently, classic conversion formulas for sensors, such as Topp’s empirical relationship [35], are inapplicable to high-salinity environments [36,37]. Therefore, each sensor type should be specifically calibrated for different soil salinity levels instead of applying factory calibration formulas [38,39] to improve their soil moisture measurement accuracy [38,40,41].

This study aimed to evaluate the reliability of eight mainstream soil moisture sensors under four (low, medium, high and extremely high) salinity levels. Specific objectives were to (1) evaluate the performance of eight different sensors under multiple salinity levels and determine the effective measurement range of soil water content, and (2) conduct specific calibrations and establish calibration formulas within each sensor’s effective measurement range for multiple salinity levels.

## 2. Materials and Methods

### 2.1. Sensors

Eight mainstream soil moisture sensors were selected for comparative analysis in laboratory soil column experiments, including TDR315L, TDR315H, TDR305H, Teros12, 5TE, EC-5, Hydra-probe II and CS655. The selected sensors were used in consistency tests with two sensors of the same type in homogeneous soils before the laboratory experiment. EC-5 outputs the voltage value while other sensors output the apparent dielectric permittivity. The soil moisture formulas of eight mainstream soil moisture sensors are listed in Table 1, where TDR315H, TDR315L, TDR305H, CS655 and 5TE sensors adopt the classical Topp’s equation [35]:

(1) $$\mathrm{VWC}=4.3\times 10^{-6}{\varepsilon_{a}}^{3}-5.5\times 10^{-4}{\varepsilon_{a}}^{2}+2.92\times 10^{-2}\varepsilon_{a}-5.3\times 10^{-2}$$

where VWC is the volumetric water content(cm^3^·cm^−3^) and ε_a_ is the apparent dielectric permittivity (-). According to the bulk electrical conductivity measurement range of eight kinds of sensors, 5TE and Teros12 might perform well in high-salinity environments because the bulk electrical conductivity thresholds could reach 20 dS·m^−1^, followed by Hydra-probe II, TDR305H, CS655, TDR315L and TDR315H.

### 2.2. Methods

We collected the original high-salinity soil sample in a salt-alkali land of the Dongling Reclamation Area in Rudong County, Jiangsu Province, China, very close to the Yellow Sea. Based on the background investigation, the salinity of the top 30 cm of soil in this area was extremely high. Considering the convenience of collection, we excavated to 10 cm depth and collected soil sample from to 0–10 cm depth. The soil sample was dried and then screened to <2 mm to remove organic residues. The texture of the soil was silty loam, with average contents of sand, silt and clay being 38.58%, 55.58%, and 5.84%, respectively (Table 2). The dry bulk density was about 1.25g·cm^−3^, and the saturated soil water content was 0.46 cm^3^·cm^−3^. The salt content was 6.8 g·kg^−1^ and the organic matter content was 3.34 g·kg^−1^. The 1:5 soil water extract electrical conductivity (EC_1:5_) was easy and fast to measure even in the field and can be appraised soil salinity effectively [42,43]. In order to assess the salinity level clearly, we also measured this variable. The EC_1:5_ of the soil sample was 3.0 dS·m^−1^.

Salt-leaching processes were performed using the original soil samples of 6.8 g·kg^−1^ to obtain three lower salinity soil samples; the EC_1:5_ values were 1.5, 1.0 and 0.75 dS·m^−1^. Therefore, the EC_1:5_ of four designed soil salinity levels were 3.0. 1.5, 1.0 and 0.75 dS·m^−1^, the corresponding soil salt contents were 6.8, 5.2, 3.6 and 3.0 g·kg^−1^, respectively (Table 3). The salinity was classified into four levels, i.e., extremely high, high, moderate and low, which referred to the classification of EC_1:5_ in Ismayilov’s work [44]. The dry bulk densities and saturated soil water contents of these three low-salinity soil samples were the same as those of the original soil samples.

**Figure 1:**
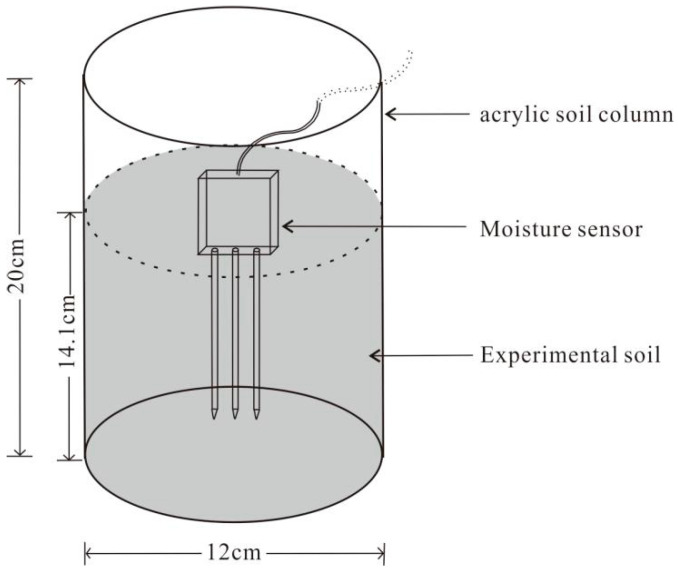
Experimental schematic diagram.

We first prepared a Level 2 salinity level soil sample (EC_1:5_ = 1.5 dS·m^−1^) using the original soil samples via the salt-leaching processes. Soil samples (10 kg) were placed in a large bucket, and deionised water was poured into the bucket until the ponding water depth was 30 cm above the soil surface. The soil and water were stirred thoroughly and then left to stand for 24 h. The ponding saline water above the soil surface was removed with a siphon so that the salt content of the soil was reduced. We took a small portion (about 100 g) of the soil sample and dried it at 105 °C. The EC values of the 1:5 soil water extract of the deionised water and dried soil sample were measured using a HI 98311 electrical conductivity metre (HANNA Instrument, Woonsocket, RI, USA) with an EC range from 0 to 3999 μS·cm^−1^. If the measured EC_1:5_ was larger than 1.5 dS·m^−1^, more deionised water was added into the bucket, and the salt-leaching process, as mentioned above, was repeated until the EC_1:5_ of the dried soil sample reached 1.5 dS·m^−1^. Then, we took other original soil samples to prepare the Level 3 and Level 4 salinity soil samples in order. The soil samples of the designed salinity levels were dried again before testing. Subsequently, soil samples at each salinity level were packed into lidless acrylic cylinders with an internal diameter of 12 cm and an inner height of 20 cm, as shown in Figure 1.

To ensure that all sensor probes could be fully inserted into the soil, the soil sample height and volume were set at 14.1 cm and 1.6 L, respectively. Based on the measured dry bulk density of the soil sample, the weight of the soil sample in the cylinder was about 2.0 kg. Six soil moisture levels (i.e., 0.20, 0.25, 0.30, 0.35, 0.40, and 0.45 cm^3^·cm^−3^) were designed for measurement. We added a specified volume of deionised water into the soil column to reach a soil moisture level, stirred, and then compacted the soil to the original height of 14.1 cm. Three parallel samples were set up. The temperature of the laboratory was maintained at about 25 °C. In order to ensure the water and salt were equally distributed in the soil column, a portable in situ soil conductivity metre, CTS 50C EC metre (Spectrum, Aurora, IL, USA), was used to measure the soil electrical conductivity at nine positions at a 10 cm depth in the soil column. The difference in the measured electrical conductivities should not exceed 0.2 dS·m^−1^. The eight types of sensors were successively inserted into the soil column to measure the soil parameters, and we repeated the measurement three times for each soil moisture sensor. The variables were acquired via a CR300 data logger (Campbell Scientific, Logan, UT, USA).

In this study, the calibration process was based on the traditional method, establishing new calibration equations using the raw output values (dielectric constant and voltage) from the sensors and the actual VWCs in the soil column. The equations were developed using the Microsoft Excel 2017 Regression Analysis tool. Generally, the calibrated equations should follow the formats of the original conversion equations at different salinity levels. However, if there was clearly a more suitable fitting function, we chose the better one.

### 2.3. Performance Evaluation Criteria

To evaluate the accuracy of the measurement of soil water content and the various proposed calibration equations, the mean absolute error (MAE) and root mean square error (RMSE) were selected [45]. The coefficient of determination (R^2^) was used to evaluate the degree of fit of the calibration equations. The specific calculation formulas are as follows:

(2) $$MAE=\frac{\sum_{i=1}^{n}\left|P_{i}-O_{i}\right|}{n}$$

(3) $$RMSE=\sqrt{\frac{\sum_{i=1}^{n}{\left(P_{i}-O_{i}\right)}^{2}}{n}}$$

(4) $$R^{2}=1-\frac{\sum_{i=1}^{n}{\left(P_{i}-O_{i}\right)}^{2}}{\sum_{i=1}^{n}{\left(P_{i}-\bar{P}\right)}^{2}}$$

where i is the data set index, n is the sample size, $P_{i}$ and O_i_ are the ith data set’s measured and reference values, respectively, and $\bar{P}$ is the average measured value.

## 3. Results

### 3.1. Analysis of Measurement Results of Each Sensor

#### 3.1.1. Qualitative Analysis

**Figure 2:**
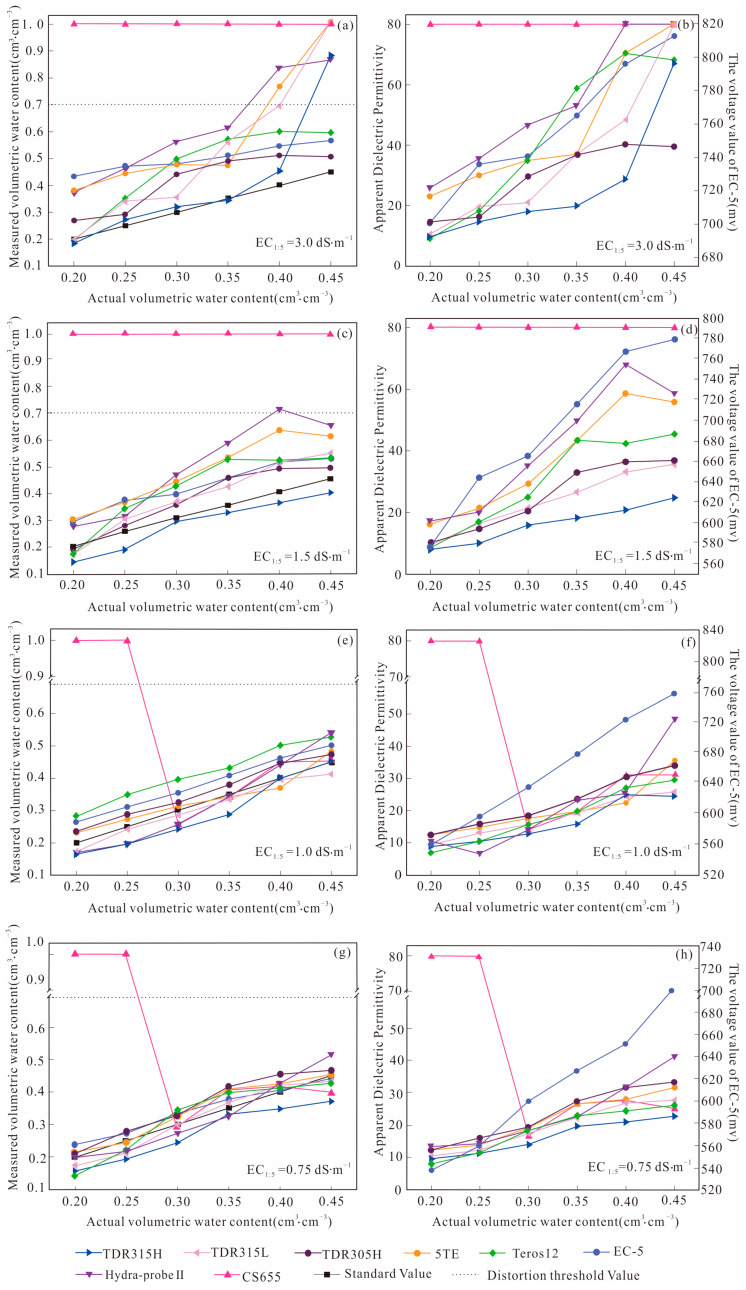
Comparison of measured VWCs and original output values of eight SMSs: (**a**) measured and actual VWCs at EC_1:5_ = 3.0 dS·m^−1^; (**b**) measured apparent dielectric permittivity and actual VWCs at EC_1:5_ = 3.0 dS·m^−1^; (**c**) measured and actual VWCs at EC_1:5_ = 1.5 dS·m^−1^; (**d**) measured apparent dielectric permittivity and actual VWCs at EC_1:5_ = 1.5 dS·m^−1^; (**e**) measured and actual VWCs at EC_1:5_ = 1.0 dS·m^−1^; (**f**) measured apparent dielectric permittivity and actual VWCs at EC_1:5_ = 1.0 dS·m^−1^; (**g**) measured and actual VWCs at EC_1:5_ = 0.75 dS·m^−1^; (**h**) measured apparent dielectric permittivity and actual VWCs at EC_1:5_ = 0.75 dS·m^−1^.

The measured VWCs and raw output values of the different sensors under the four soil salinity levels are shown in Figure 2.

Based on the soil’s physical characteristics, the maximum VWCs of the natural soil were about 0.70 cm^3^·cm^−3^ [46]. Therefore, the distortion threshold of the measured VWCs was set at 0.70 cm^3^·cm^−3^. It can be observed from Figure 2a,c that the measured results of the CS655 sensor showed severe distortion under 6.8 and 5.2 g·kg^−1^ salinity levels. However, distortion could only be observed when the actual VWC was below 0.30 cm^3^·cm^−3^ under 3.6 and 3.0 g·kg^−1^ salinity levels using the CS655 sensor, as shown in Figure 2e,g.

The measured VWCs of the TDR315H sensor were very similar to the standard values when the VWCs were less than 0.45 cm^3^·cm^−3^ under a salinity level of 1. The measured values exceeded the distortion threshold as the actual VWC grew to 0.45 cm^3^·cm^−3^ (Figure 2a). Although the measured VWCs of TDR315L, Hydra-probe II, and 5TE gave significant overestimations when the actual VWCs were below 0.40 cm^3^·cm^−3^, their variation tendency was similar to that of the standard values. When the actual VWCs exceeded 0.40 cm^3^·cm^−3^, the measured values of these three sensors rose rapidly and exceeded the distortion threshold. In contrast, TDR305H, Teros12 and EC-5 showed no distortion within the total soil moisture range under study.

All sensors, except CS655, performed better at salinity Level 2 than at salinity Level 1. Obviously, the results of six sensors, i.e., TDR315L, TDR305H, Teros12, 5TE, EC-5 and Hydra-probe II, were overestimated compared to the standard values (see Figure 2c). Moreover, the overestimation degrees of Hydra-probe II and 5TE intensified as the actual VWCs increased. Quite unexpectedly, the results of TDR315H were continuously underestimated compared to the standard values. Distortion only occurred for the Hydra-probe II sensor when the actual VWC was 0.40 cm^3^·cm^−3^. These sensors’ variation trend was the same as that of the standard values at salinity Level 3, as shown in Figure 2e. TDR315L, TDR305H, 5TE and Hydra-probe II sensors performed reasonably well at this salinity level. The results of TDR315H were underestimated when the actual VWC was below 0.40 cm^3^·cm^−3^, while Teros12 and EC-5 overestimated the values within the total soil moisture range. All the sensors performed well at the lowest salinity level of 4, as shown in Figure 2g. The average errors of most sensors were below 0.05 cm^3^·cm^−3^. The results of TDR315H were consistently underestimated, while the measured VWCs of EC-5 were always overestimated.

The measured VWCs of all sensors were obtained by converting the raw output parameters, i.e., the apparent dielectric permittivity and voltage. Therefore, the variation in the raw output parameters of all sensors was consistent with that of the corresponding measured VWCs, which can be seen in Figure 2b,d,f,h. Although the raw data were the same, the different conversion formulas used in these sensors strongly influenced the final VWCs. As the threshold of the measured apparent dielectric permittivity was 80, when the measured apparent dielectric permittivity was close to 80, the measured VWCs might exceed the distortion threshold. The measured apparent dielectric permittivity values of the TDR series, i.e., 305H, 315L and 315H, were the smallest among all the sensors in relatively high-salinity levels 1 and 2, suggesting that the raw parameters of these sensors should be adjusted specifically for high-salinity environments.

Teros12, TDR305H and EC-5 sensors did not exceed the distortion threshold (0.70 cm^3^·cm^−3^) under the four designed salinity levels. Notably, when the actual VWC exceeded 0.40 cm^3^·cm^−3^ at salinity Level 1, the measured VWCs of Teros12 and TDR305H declined abnormally while the measured values of EC-5 increased as expected. The measured apparent dielectric permittivity of the former two sensors seemed insensitive to the increase in soil water content at that stage. Therefore, the measured VWCs of Teros12 and TDR305H were no longer reliable when the actual VWC was 0.45 cm^3^·cm^−3^. Compared to the features of the distortion type mentioned above, such a distortion phenomenon was quite different. Accordingly, we classified the observed distortion phenomena into two types. The first was mutational distortion, i.e., as the actual VWC increased, the measured apparent dielectric permittivity increased sharply and was close to 80. Therefore, the measured VWCs may exceed the distortion threshold. Such distortion occurred in the TDR315L, TDR315H, Hydra-probe II, and 5TE sensors. The second type of distortion was named insensitive distortion, i.e., as the actual VWC increased, the measured apparent dielectric permittivity declined, which occurred in Teros12 and TDR305H at salinity Level 1, as well as in Hydra-probe II, 5TE, Teros12 and TDR305H at salinity Level 2.

**Figure 3:**
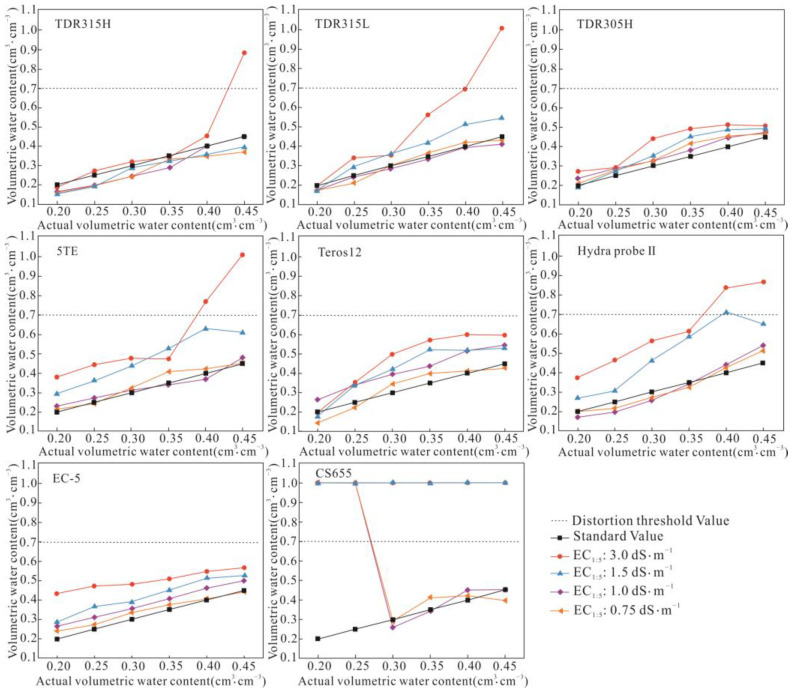
Soil water content measured by eight soil moisture sensors at four salinity levels.

As the salt content increased, the overestimation degree of all sensors increased, as shown in Figure 3. Higher soil moisture content also intensified the degree of overestimation at high and extremely high-salinity levels 1 and 2. Overall, EC-5 performed best at the four salinity levels, with the measured values showing a good linear trend compared to the standard values. The TDR series, i.e., 305H, 315L, and 315H, 5TE, and Teros12, showed good performance, while CS655 performed the worst.

This study adopted the mean absolute error (MAE) and root mean square error (RMSE) to evaluate the overall degree of overestimation of the effective measured VWCs of the eight selected sensors, as shown in Table 4.

At salinity Level 1, the MAE and RMSE values of the seven sensors were in the range of 0.024–0.229 and 0.029–0.232 cm^3^·cm^−3^. At salinity Level 2, these were 0.040–0.152 and 0.043–0.160 cm^3^·cm^−3^, reaching 0.018–0.090 and 0.021–0.090 cm^3^·cm^−3^ at salinity Level 3. Finally, at the lowest salinity Level 4, the MAE range was 0.020–0.051 cm^3^·cm^−3^, and the RMSE range was 0.023–0.054 cm^3^·cm^−3^. Generally, the lower the salinity Level, the lower the MAE and RMSE values, indicating higher accuracy. The results showed that the MAE and RMSE values of the TDR315H and TDR305H sensors were the smallest among the eight sensors. MAE and RMSE values of TDR315L exceeded 0.10 cm^3^·cm^−3^ at the extremely high-salinity Level 1. However, as the soil salinity decreased, the values of the two indicators were below 0.10 cm^3^·cm^−3^. The RMSE values of Hydra-probe II, EC-5, 5TE, and Teros12 sensors exceeded 0.10 cm^3^·cm^−3^ at salinity levels 1 and 2. These four sensors’ RMSE and MAE values dropped below 0.10 cm^3^·cm^−3^ only at the lowest salinity Level 4. The soil salt content significantly impacted the stability and accuracy of these sensors.

#### 3.1.2. Calibration at Different Salinity Levels

**Figure 4:**
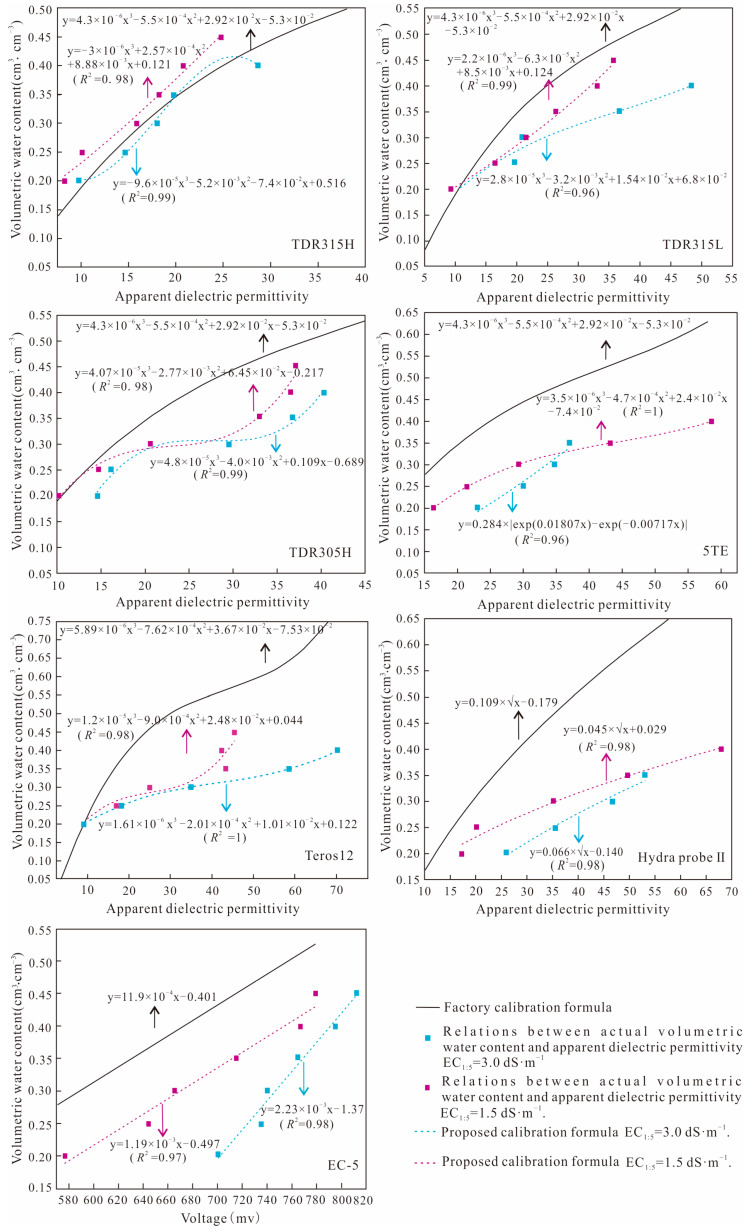
The proposed calibration formulas of soil moisture sensors at salinity levels EC_1:5_ = 3.0 dS·m^−1^ and EC_1:5_ = 1.5 dS·m^−1^.

**Figure 5:**
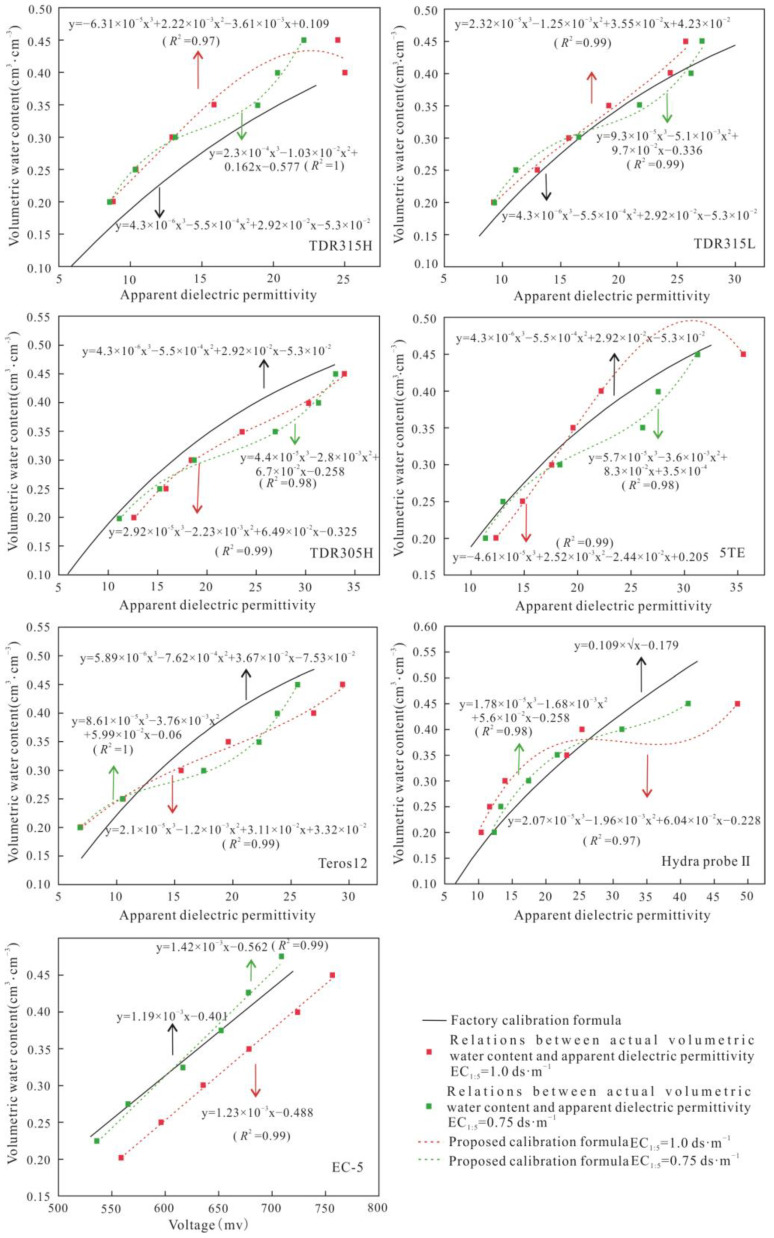
The proposed calibration formulas of soil moisture sensors at salinity levels EC_1:5_ = 1.0 dS·m^−1^ and EC_1:5_ = 0.75 dS·m^−1^.

Given the overestimation problem of the measured VWCs by these soil moisture sensors in high-salinity environments, this study derived empirical formulas linking the actual VWC values with the raw output values of seven sensors (excluding CS655) below the distortion thresholds. TDR315H, TDR315L, TDR305H, Teros12, EC-5 and Hydra-probe Ⅱ were calibrated based on the corresponding formats of the original conversion formulas at different salinity levels. An exponential function with a higher degree of fitting was chosen for 5TE. The details are shown in Figure 4 and Figure 5.

The parameters R^2^ and RMSE were used to quantify the accuracy of the new calibration formula for each soil moisture sensor below the distortion thresholds, as shown in Table 5. After calibration, the R^2^ values of the measured VWCs of each sensor ranged from 0.96 to 1.00, while the RMSE values varied from 0.001 to 0.013 cm^3^·cm^−3^. Compared with the factory conversion formulas, the proposed formulas greatly improved the measurement accuracy of the VWCs of each sensor at high-salinity levels.

## 4. Discussion

In this study, the performance of eight mainstream soil moisture sensors, i.e., TDR315H, TDR315L, TDR305H, 5TE, Teros12, Hydra-probe Ⅱ, EC-5 and CS655, were compared at four soil salinity levels, analysing the overestimation degree of measured VWCs at each salinity level. The overestimation degree of all sensors increased as the salt content of the soil increased. Actually, two types of distortion phenomena, i.e., mutational distortion and insensitive distortion, were caused by the extremely high-salinity environment and high soil moisture conditions. These two factors would dramatically influence the transmission of electromagnetic waves and cause raw data, i.e., apparent dielectric permittivity, to be overestimated or irregularly declined. Although the official electrical conductivity thresholds of 5TE and Teros12 could reach 20 dS·m^−1^, this parameter failed to reflect the performance of the measured VWCs in the corresponding high-salinity soils. Soil salinity definitely plays a crucial role in affecting the accuracy of VWC measurements.

We proposed calibration formulas for each sensor at four salinity levels within the corresponding distortion thresholds. The calibration process was based on the traditional method, establishing new conversion formulas using the raw output values (dielectric constant and voltage) and the actual VWCs in the soil column. The precision of the proposed calibration formulas reached ±0.02 cm^3^·cm^−3^ at all salinity levels under study. Therefore, manufacturers of soil moisture sensors should substantiate and propose specific conversion formulas for different salinity levels rather than provide a single recommended formula for a soil type without considering the effect of salinity. Appropriate calibration can greatly improve the measurement accuracy of soil moisture sensors [41,47,48]. According to the quantitative analysis of the overestimation degree of the measured VWCs at these salinity levels, a simpler calibration principle might be applied, i.e., subtracting a constant term (MAE) in the factory-calibrated formula at a certain salinity level, which was used in the calibration of CS616 in Yang’s work [45].

Several researchers [23] have also proved that soil texture, especially clay content, greatly influences the performance of soil moisture sensors. Although we quantified the measurement performance of these eight soil moisture sensors in atypical soil, i.e., silty loam, of China’s eastern coastal area, the performance of these selected soil moisture sensors in other salinity soil types at various soil salinity levels is still unknown, which needs to be studied in the future [13]. Moreover, other mainstream or newly developed soil moisture sensors should also be included in such laboratory tests at different salinity levels to provide a more complete view of the performance of each sensor. Furthermore, considering the short period of laboratory tests, a life cycle assessment study of those soil moisture sensors when they are buried for a long time in high-salinity soils in the field is also required. We took great care to ensure homogeneous soil conditions during the different sensor measurements. However, small variabilities in the bulk density and imperfect mixing of soil and added water between the different wetting steps can result in larger errors in dielectric permittivity measurements using the sensors. However, there is still a need to improve this experimental approach.

## 5. Conclusions

This study performed laboratory soil column tests, showing that soil salinity significantly impacted the measurement accuracy of eight mainstream soil moisture sensors. As the salt content increased, the overestimation degree of all sensors increased. Only EC-5 exhibited no distortion at high-salinity levels, with the measured values showing a good linear trend compared to the standard values. Mutational distortion of the measured apparent dielectric permittivity occurred in TDR315L, TDR315H, Hydra-probe II, and 5TE at EC_1:5_ = 3.0 dS·m^−1^. Insensitive distortion of the measured apparent dielectric permittivity occurred in Teros12 and TDR305H at EC_1:5_ = 3.0 dS·m^−1^ as well as in Teros12, TDR305H, 5TE, and Hydra-probe II at EC_1:5_ = 1.5 dS·m^−1^. All tested sensors performed reasonably well at EC_1:5_ ≤ 1.0 dS·m^−1^.

Seven sensors (excluding CS655) were calibrated within the distortion threshold. The soil moisture accuracy using the calibrated formulas could reach ±0.02 cm^3^·cm^−3^. At EC_1:5_ ≤ 1.0 dS·m^−1^, most sensors in this study could be applied with the factory calibration formulas.

After calibration, the TDR series, EC-5, 5TE and Teros12 were recommended for EC_1:5_ > 1.0 dS·m^−1^. For extremely high soil salinity levels, the TDR series and EC-5 may be the best options.

## Figures and Tables

**Table 1:** Parameters of eight soil moisture sensors in this study.

Sensor Model	Manufacturer	Measuring Technique	Measured Parameter	Conversion Equation of VWC	Bulk Electrical Conductivity Range (dS·m^−1^)
TDR315H	Acclima, Meridian, ID, USA	TDR	ε_a_, EC_b_, T	VWC = 4.3 × 10^−6^ε_a_^3^ − 5.5 × 10^−4^ε_a_^2^ + 2.92 × 10^−2^ε_a_ − 5.3 × 10^−2^	0~5
TDR315L	Acclima, Meridian, ID, USA	TDR	ε_a_, EC_b_, T	VWC = 4.3 × 10^−6^ε_a_^3^ − 5.5 × 10^−4^ε_a_^2^ + 2.92 × 10^−2^ε_a_ − 5.3 × 10^−2^	0~5
TDR305H	Acclima, Meridian, ID, USA	TDR	ε_a_, EC_b_, T	VWC = 4.3 × 10^−6^ε_a_^3^ − 5.5 × 10^−4^ε_a_^2^ + 2.92 × 10^−2^ε_a_ − 5.3 × 10^−2^	0~10
CS655	Campbell Scientific, Logan, UT, USA	TLO	ε_a_, EC_b_, T	VWC = 4.3 × 10^−6^ε_a_^3^ − 5.5 × 10^−4^ε_a_^2^ + 2.92 × 10^−2^ε_a_ − 5.3×10^−2^	0~8
5TE	METER Group, Pullman, WA, USA	FDR	ε_a_, EC_b_, T	VWC = 4.3 × 10^−6^ε_a_^3^ − 5.5 × 10^−4^ε_a_^2^ + 2.92 × 10^−2^ε_a_ − 5.3 × 10^−2^	0~23
EC-5	METER Group, Pullman, WA, USA	FDR	Voltage	VWC = (11.9 × 10^−4^)(mv) − 0.401	/
Teros12	METER Group, Pullman, WA, USA	TDR	ε_a_, EC_b_, T	VWC = 5.89 × 10^−6^ε_a_^3^ − 7.62 × 10^−4^ε_a_^2^ + 3.67 × 10^−2^ε_a_ − 7.53 × 10^−2^	0~20
Hydra-probe II	Stevens Water, Portland, OR, USA	TDR	ε_a_, EC_b_, T	VWC = 0.109√ε_a_ − 0.179	0~15

Note: T is temperature; ε_a_ is apparent dielectric permittivity; EC_b_ is bulk electrical conductivity; mv is voltage value; VWC is volumetric water content; and / is null value.

**Table 2:** Physical properties of the soil samples.

Texture Class USDA	Sand (% weight)	Slit (% weight)	Clay (% weight)	Dry Bulk Density (g·cm^−3^)	Saturated Soil Water Content (cm^3^·cm^−3^)	Total Salt Content (g·kg^−1^)	Organic Matter Content (g·kg^−1^)
Silty loam	38.58	55.58	5.84	1.25	0.46	6.8	3.34

**Table 3:** Salinity classification of the tested soil samples.

Number	EC_1:5_ (dS·m^−1^)	Total Salt Content (g·kg^−1^)	Salinity Classification
1	3.00	6.8	Extremely high
2	1.50	5.2	High
3	1.00	3.6	Moderate
4	0.75	3.0	Low

**Table 4:** Comparative analysis of measured soil moisture values obtained via the original formulas and oven-drying method.

Sensor Model	1-Extremely High		2-High		3-Moderate		4-Low	
	MAE	RMSE	MAE	RMSE	MAE	RMSE	MAE	RMSE
	cm^3^·cm^−3^		cm^3^·cm^−3^		cm^3^·cm^−3^		cm^3^·cm^−3^	
TDR315H	0.024	0.029	0.040	0.043	0.035	0.043	0.051	0.054
TDR315L	0.132	0.170	0.070	0.076	0.018	0.021	0.020	0.023
TDR305H	0.101	0.109	0.052	0.062	0.033	0.034	0.034	0.039
5TE	0.170	0.172	0.152	0.160	0.023	0.025	0.022	0.028
Teros12	0.146	0.167	0.101	0.115	0.090	0.090	0.035	0.039
Hydra-probe II	0.229	0.232	0.132	0.150	0.044	0.050	0.029	0.035
EC-5	0.177	0.181	0.098	0.099	0.058	0.058	0.023	0.026
CS655	-	-	-	-	-	-	-	-

Note: - means out of range.

**Table 5:** Comparative analysis of soil moisture values obtained via the proposed calibration formulas and the oven-drying method.

Sensor Model	Effective VWC Range (cm^3^·cm^−3^)	Calibration Formula (EC_1:5_ = 3.0 dS·m^−1^)	R^2^	MAE (cm^3^·cm^−3^)	RMSE (cm^3^·cm^−3^)
TDR315H	0.20~0.40	y = −9.6 × 10^−5^ε_a_^3^ − 5.2 × 10^−3^ε_a_^2^ − 7.4 × 10^−2^ε_a_ + 0.516	0.99	0.005	0.006
TDR315L	0.20~0.40	y = 2.8 × 10^−6^ε_a_^3^ − 3.2 × 10^−6^ε_a_^2^ + 1.54 × 10^−2^ε_a_ + 0.068	0.96	0.009	0.013
TDR305H	0.20~0.40	y = 4.8 × 10^−5^ε_a_^3^ − 4.0 × 10^−3^ε_a_^2^ + 0.109ε_a_ − 0.689	0.99	0.007	0.007
5TE	0.20~0.35	y = 0.284 × [exp(0.01807ε_a_) − exp(−0.00717ε_a_)]	0.96	0.011	0.011
Teros12	0.20~0.40	y = 1.61 × 10^−6^ε_a_^3^ − 2.01 × 10^−4^ε_a_^2^ + 1.01 × 10 ^− 2^ε_a_ + 0.122	1.00	0.001	0.001
Hydra-probe II	0.20~0.35	y = 0.66√ε_a_ − 0.140	0.98	0.007	0.008
EC-5	0.20~0.45	y = 2.23 × 10^−3^mv − 1.37	0.98	0.011	0.013
Sensor Model	Effective VWC Range (cm^3^·cm^−3^)	Calibration Formula (EC_1:5_ = 1.5 dS·m^−1^)	R^2^	MAE (cm^3^·cm^−3^)	RMSE (cm^3^·cm^−3^)
TDR315H	0.20~0.45	y = −3.0 × 10^−6^ε_a_^3^ + 2.57 × 10^−4^ε_a_^2^ + 8.88 × 10^−3^ε_a_ + 0.121	0.98	0.004	0.006
TDR315L	0.20~0.45	y = 2.2 × 10^−6^ε_a_^3^ − 6.3 × 10^−5^ε_a_^2^ + 8.5 × 10^−3^ε_a_ + 0.124	0.98	0.009	0.013
TDR305H	0.20~0.45	y = 4.07 × 10^−5^ε_a_^3^ − 2.77 × 10^−3^ε_a_^2^ + 6.45 × 10^−2^ε_a_ − 0.217	0.98	0.007	0.007
5TE	0.20~0.40	y = 3.5 × 10^−6^ε_a_^3^ − 4.7 × 10^−4^ε_a_^2^ + 2.4 × 10^−2^ε_a_ − 0.074	1.00	0.005	0.011
Teros12	0.20~0.45	y = 1.2 × 10^−5^ε_a_^3^ − 9.0 × 10^−4^ε_a_^2^ + 2.48 × 10^−2^ε_a_ + 0.044	0.98	0.020	0.027
Hydra-probe II	0.20~0.40	y = 0.045√ε_a_ + 0.029	0.98	0.007	0.007
EC-5	0.20~0.45	y = 1.19 × 10^−3^mv − 0.497	0.97	0.011	0.013
Sensor Model	Effective VWC Range (cm^3^·cm^−3^)	Calibration Formula (EC_1:5_ = 1.0 dS·m^−1^)	R^2^	MAE (cm^3^·cm^−3^)	RMSE (cm^3^·cm^−3^)
TDR315H	0.20~0.45	y = −6.31 × 10^−5^ε_a_^3^ + 2.22 × 10^−3^ε_a_^2^ − 3.61 × 10^−3^ε_a_ + 0.109	0.97	0.012	0.014
TDR315L	0.20~0.45	y = 2.32 × 10^−5^ε_a_^3^ − 1.25 × 10^−3^ε_a_^2^ + 3.55 × 10^−2^ε_a_ + 0.0423	0.99	0.008	0.010
TDR305H	0.20~0.45	y = 2.92 × 10^−5^ε_a_^3^ − 2.23 × 10^−3^ε_a_^2^ + 6.49 × 10^−2^ε_a_ − 0.325	0.99	0.004	0.005
5TE	0.20~0.45	y = −4.61 × 10^−5^ε_a_^3^ + 2.52 × 10^−3^ε_a_^2^ − 2.44 × 10^−2^ε_a_ + 0.205	0.99	0.002	0.003
Teros12	0.20~0.45	y = 2.1 × 10^−5^ε_a_^3^ − 1.2 × 10^−3^ε_a_^2^ + 3.11 × 10^−2^ε_a_ + 0.0332	0.99	0.006	0.007
Hydra-probe II	0.20~0.45	y = 2.07 × 10^−5^ε_a_^3^ − 1.96 × 10^−3^ε_a_^2^ + 6.04 × 10^−2^ε_a_ − 0.228	0.97	0.012	0.015
EC-5	0.20~0.45	y = 1.23 × 10^−3^mv − 0.488	0.99	0.003	0.003
Sensor Model	Effective VWC Range (cm^3^·cm^−3^)	Calibration Formula (EC_1:5_ = 0.75 dS·m^−1^)	R^2^	MAE (cm^3^·cm^−3^)	RMSE (cm^3^·cm^−3^)
TDR315H	0.20~0.45	y = 2.3 × 10^−4^ε_a_^3^ − 1.03 × 10^−2^ε_a_^2^ + 0.162ε_a_ − 0.577	1.00	0.005	0.006
TDR315L	0.20~0.45	y = 9.3 × 10^−5^ε_a_^3^ − 5.1 × 10^−3^ε_a_^2^ + 9.7 × 10^−2^ε_a_ − 0.336	0.99	0.008	0.009
TDR305H	0.20~0.45	y = 4.4 × 10^−5^ε_a_^3^ − 2.8 × 10^−3^ε_a_^2^ + 6.7 × 10^−2^ε_a_ − 0.258	0.98	0.006	0.007
5TE	0.20~0.45	y = 5.7 × 10^−5^ε_a_^3^ − 3.6 × 10^−3^ε_a_^2^ + 8.3 × 10^−2^ε_a_ + 3.5 × 10^−4^	0.98	0.009	0.011
Teros12	0.20~0.45	y = 8.61 × 10^−5^ε_a_^3^ − 3.76 × 10^−3^ε_a_^2^ + 5.99 × 10^−2^ε_a_ − 0.06	1.00	0.003	0.004
Hydra-probe II	0.20~0.45	y = 1.78 × 10^−5^ε_a_^3^ – 168 × 10^−3^ε_a_^2^ + 5.6 × 10^−2^ε_a_ − 0.258	0.98	0.006	0.008
EC-5	0.20~0.50	y = 1.42 × 10^−3^mv − 0.562	0.99	0.008	0.009

## Data Availability

Availability upon request.
